# Supplementary material for: The Construction and Exploration of a Comprehensive MicroRNA Centered Regulatory Network in Foxtail Millet (Setaria italica L.)
Source: Front Plant Sci. 2022 May 6;13:848474. doi: 10.3389/fpls.2022.848474 (PMC9121102; doi:10.3389/fpls.2022.848474)
Supplement: Supplementary file 3 [file Data_Sheet_3.zip › Sit-miR172b_Seita.7G263000.1_1947_TPlot.pdf]

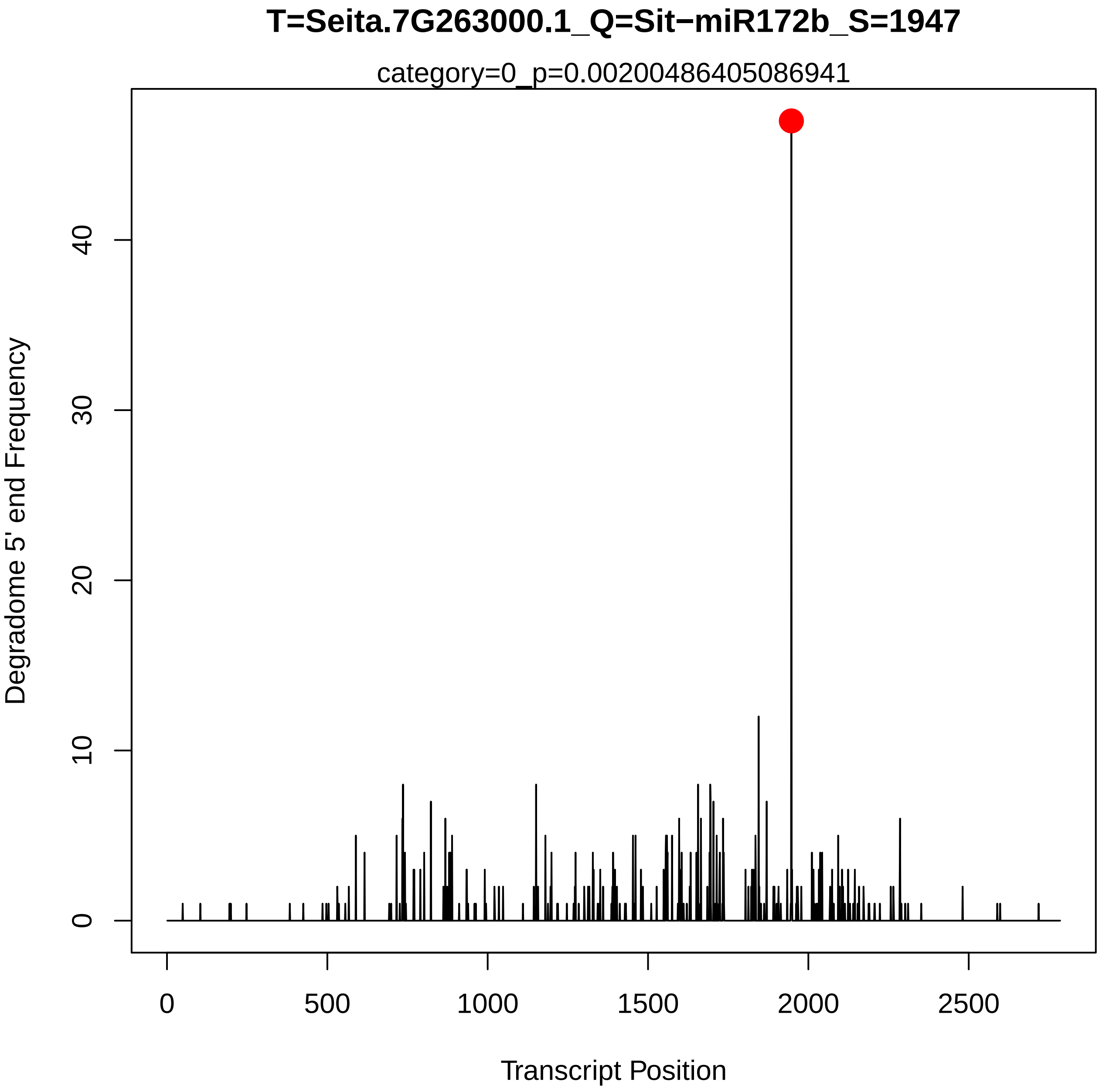

T=Seita.7G263000.1_Q=Sit−miR172b_S=1947
category=0_p=0.00200486405086941
Degradome 5' end Frequency
0
10
20
30
40
0
500
1000
1500
2000
2500
Transcript Position
